# Diversity and prevalence of gastrointestinal parasites in seven non-human primates of the Taï National Park, Côte d’Ivoire

**DOI:** 10.1051/parasite/2015001

**Published:** 2015-01-27

**Authors:** Roland Yao Wa Kouassi, Scott William McGraw, Patrick Kouassi Yao, Ahmed Abou-Bacar, Julie Brunet, Bernard Pesson, Bassirou Bonfoh, Eliezer Kouakou N’goran, Ermanno Candolfi

**Affiliations:** 1 Unité de Formation et de Recherche Biosciences, Université Félix Houphouët Boigny 22 BP 770 Abidjan 22 Côte d’Ivoire; 2 Centre Suisse de Recherches Scientifiques en Côte d’Ivoire 01 BP 1303 Abidjan 01 Côte d’Ivoire; 3 Department of Anthropology, Ohio State University, 4064 Smith Laboratory 174 West 18th Avenue Columbus Ohio 43210 USA; 4 Laboratoire de Parasitologie et de Mycologie Médicale, Plateau Technique de Microbiologie, Hôpitaux Universitaires de Strasbourg 1 rue Koeberlé 67000 Strasbourg France; 5 Laboratoire de Parasitologie, Faculté de Pharmacie, Université de Strasbourg 74 route du Rhin 67401 Illkirch cedex France; 6 Institut de Parasitologie et de Pathologie Tropicale, EA 7292, Fédération de Médecine Translationnelle, Université de Strasbourg 3 rue Koeberlé 67000 Strasbourg France

**Keywords:** Gastrointestinal parasites, Non-human primates, Prevalence, Côte d’Ivoire

## Abstract

Parasites and infectious diseases are well-known threats to primate populations. The main objective of this study was to provide baseline data on fecal parasites in the cercopithecid monkeys inhabiting Côte d’Ivoire’s Taï National Park. Seven of eight cercopithecid species present in the park were sampled: *Cercopithecus diana*, *Cercopithecus campbelli*, *Cercopithecus petaurista*, *Procolobus badius*, *Procolobus verus*, *Colobus polykomos*, and *Cercocebus atys*. We collected 3142 monkey stool samples between November 2009 and December 2010. Stool samples were processed by direct wet mount examination, formalin-ethyl acetate concentration, and MIF (merthiolate, iodine, formalin) concentration methods. Slides were examined under microscope and parasite identification was based on the morphology of cysts, eggs, and adult worms. A total of 23 species of parasites was recovered including 9 protozoa (*Entamoeba coli*, *Entamoeba histolytica/dispar*, *Entamoeba hartmanni*, *Endolimax nana*, *Iodamoeba butschlii*, *Chilomastix mesnili*, *Giardia* sp., *Balantidium coli*, and *Blastocystis* sp.), 13 nematodes (*Oesophagostomum* sp., *Ancylostoma* sp., *Anatrichosoma* sp., Capillariidae Gen. sp. 1, Capillariidae Gen. sp. 2, *Chitwoodspirura* sp., *Subulura* sp., spirurids [cf *Protospirura muricola*], *Ternidens* sp., *Strongyloides* sp., *Trichostrongylus* sp., and *Trichuris* sp.), and 1 trematode (*Dicrocoelium* sp.). Diversity indices and parasite richness were high for all monkey taxa, but *C. diana*, *C. petaurista*, *C. atys*, and *C. campbelli* exhibited a greater diversity of parasite species and a more equitable distribution. The parasitological data reported are the first available for these cercopithecid species within Taï National Park.

## Abstract (fr)

Les maladies parasitaires et infectieuses sont des menaces très connues pour les populations de primates. L’objectif principal de cette étude était de fournir des données de base sur les parasites intestinaux des primates non-humains du Parc National de Taï en Côte d’Ivoire. Sept des huit espèces de cercopithécidés vivant dans le parc ont été échantillonnées : *Cercopithecus diana*, *Cercopithecus campbelli*, *Cercopithecus petaurista*, *Procolobus badius*, *Procolobus verus*, *Colobus polykomos* and *Cercocebus atys*. Nous avons collecté 3142 échantillons de selles de singes de novembre 2009 à décembre 2010. Les échantillons de selles ont été traités par la technique d’examen direct, les méthodes de concentration formol-éthyl acétate et MIF (merthiolate, iode, formol). Les lames ont été examinées au microscope et l’identification des parasites a été basée sur la morphologie des kystes, des œufs et des vers adultes. Au total, 23 espèces de parasites ont été trouvées, dont 9 protozoaires (*Entamoeba coli*, *Entamoeba histolytica/dispar*, *Entamoeba hartmanni*, *Endolimax nana*, *Iodamoeba butschlii*, *Chilomastix mesnili*, *Giardia* sp., *Balantidium coli* et *Blastocystis* sp.), 13 nématodes (*Oesophagostomum* sp., *Ancylostoma* sp., *Anatrichosoma* sp., Capillariidae Gen. sp. 1, Capillariidae Gen. sp. 2, *Chitwoodspirura* sp., *Subulura* sp., Spiruridae [cf. *Protospirura muricola*], *Ternidens* sp., *Strongyloides* sp., *Trichostrongylus* sp. et *Trichuris* sp.), et un trématode (*Dicrocoelium* sp.). L’indice de diversité et la richesse parasitaire étaient élevés pour tous les taxa de singes, mais *C. diana*, *C. petaurista*, *C. atys* and *C. campbelli* ont enregistré une plus grande diversité et une distribution plus équitable des espèces de parasites. Les données parasitologiques que nous rapportons sont les premières disponibles pour ces espèces de singes du Parc National de Taï.

## Introduction

Côte d’Ivoire’s Taï National Park (TNP) is the last remaining major intact block of primary forest in West Africa. It was declared a UNESCO World Heritage Site in 1982 due to exceptional richness in fauna and flora. Indeed, based on several criteria including species diversity, endemism, presence of rare species and/or endangered and critical habitats, the TNP is considered a priority for the conservation of mammals, birds, amphibians, and invertebrates in West Africa [13]. The park contains fauna typical of West African forests, including eight monkey species: Diana monkey (*Cercopithecus diana*), Campbell’s monkey (*Cercopithecus campbelli*), lesser spot-nosed monkey (*Cercopithecus petaurista*), putty-nosed monkey (*Cercopithecus nictitans*), red colobus monkey (*Procolobus badius*), olive colobus monkey (*Procolobus verus*), western black and white colobus monkey (*Colobus polykomos*), and sooty mangabey (*Cercocebus atys*). Since 1989, multiple aspects of behavior, ecology, and anatomy of this primate community have been subjected to investigation by members of the Taï Monkey Project (TMP) including mixed-species associations, feeding, ranging, positional behavior, vocalization, sociality, anti-predator adaptations, and conservation [44]. To date, no studies have been undertaken on the gastrointestinal parasites of monkeys in this region.

Many protozoa and helminths are known to infect primate groups [69]. Parasites and infectious diseases have become a major concern in conservation biology, in part because they can trigger or accelerate population declines [3]. Many studies have documented the gastrointestinal parasites of wild populations of African primates [18, 19, 26, 30, 40, 45, 60]. In recent years, several infectious diseases have been recorded in primate groups followed by researchers from monkey and chimpanzee projects in Taï National Park [16, 21, 32, 34, 37]. However, the diversity and abundance of gastrointestinal parasites in the cercopithecids inhabiting forests within West Africa’s Upper Guinea region have yet to be examined systematically. In this study, we identify and quantify the prevalence of gastrointestinal parasites in seven sympatric primates ranging within Côte d’Ivoire’s Taï National Park. Our report on parasite diversity constitutes the first parasite data for cercopithecid primates within this tropical region.

## Materials and methods

### Study site

**Figure 1.**
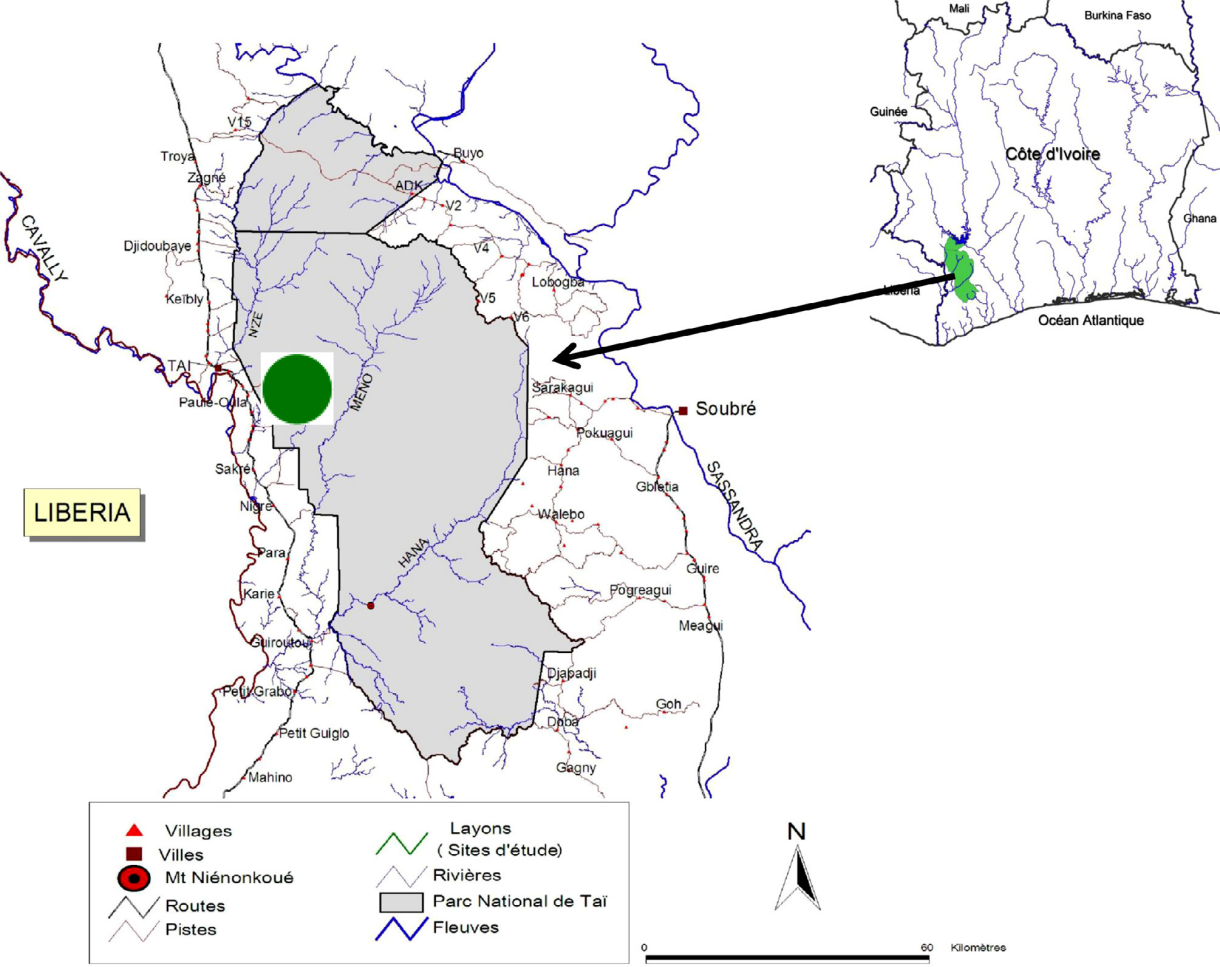
Location of the study area in Taï National Park, Côte d’Ivoire Green dot = Study area.

The study was conducted in Taï National Park in southwestern Côte d’Ivoire (6°20 N–5°10 N and 4°20 W–6°50 W). The park is the last remaining major block of primary forest in West Africa. It covers approximately 457,000 ha of rainforest and is bordered in the North by the N’zo Wildlife Reserve (79,000 ha) (Fig. 1). Annual rainfall averages 1800 mm and daily temperatures average 24 °C. The climate is characterized by four seasons: two rainy seasons (March–June and September–November) and two dry seasons (December–February and July–August). The study site has been described in detail elsewhere [42]. The seven cercopithecid taxa investigated are *Cercocebus atys*, *Cercopithecus campbelli*, *Cercopithecus diana*, *Cercopithecus petaurista*, *Colobus polykomos*, *Procolobus badius*, and *Procolobus verus*. Since 1991, these monkeys have been under continuous study in Taï National Park and at least one (in some cases three) social group of each species is fully habituated to human observers [31, 44]. With one exception, the home ranges of all monkey groups sampled are contained within the 2 km^2^ core area of the Taï Monkey Project (McGraw et al. 2007). The home range of *Cercocebus atys* is significantly larger than those of the other species and extends beyond the primary study grid. The relationships between locomotion and habitat use in these monkey taxa have been described elsewhere [42, 43].

### Field and laboratory procedures

Between November 2009 and December 2010, we collected fecal samples from all monkey taxa during dawn to dusk (06:30–18:00). Over the course of the 13 month sampling period, we collected 3142 fecal samples from the seven primate species. Table 1 provides information on the demographic composition of each monkey species. To avoid contamination, fecal samples were collected from the center of each fecal mass immediately following defecation. Immediately upon returning to the field station located adjacent to the primary study grid, samples were placed individually in 20.0 mL sterile vials in 10% formalin solution in order to avoid contamination. Each sample was studied by wet mount examination, Ritchie method modified using Formalin-ethyl acetate concentration [15, 35, 73], to diagnose helminths and protozoa; additionally, the merthiolate-iodine-formalin concentration (MIFC) technique was used for better identification of intestinal protozoa [8]. Eggs and cysts were detected under a microscope (Leica DM2000 LED) equipped with a digital camera control unit (Leica DFC450). Parasites were identified on the basis of egg color, shape, contents, size, larvae, and cysts [24, 28]. Measurements were made to the nearest 0.1 μm using a micrometer integrated into the digital camera. Representatives of each parasite were photographed.

**Table 1.** Composition of monkey groups.

	*Cercocebus atys*	*Procolobus badius*	*Colobus polykomos*	*Procolobus verus*	*Cercopithecus campbelli*	*Cercopithecus petaurista*	*Cercopithecus diana*
Adult Male	6	11	2	2	1	1	1
Adult Female	33	30	2	2	7	8	9
Subadult Male	18	3	2	2	2	3	2
Subadult Female	19	2	1	–	1	1	6
Juvenile Male	21	–	1	–	2	2	1
Juvenile Female	28	2	–	–	3	2	3
Infant	–	3	–	1	1	2	3
Total	125	51	8	7	16	19	25

### Statistical analysis

Data were entered into an Excel spreadsheet and transferred into Stata version 11 (StataCorp.; College Station, United States of America). Fisher’s exact test was used to compare parasite prevalence between monkey taxa. *P* values below 0.05 were considered as significant. The Shannon diversity index was used to measure diversity, abundance, and equitability of parasite species present in the monkey community.

## Results

### Parasite species of non-human primates in Taï forest

**Figure 2.**
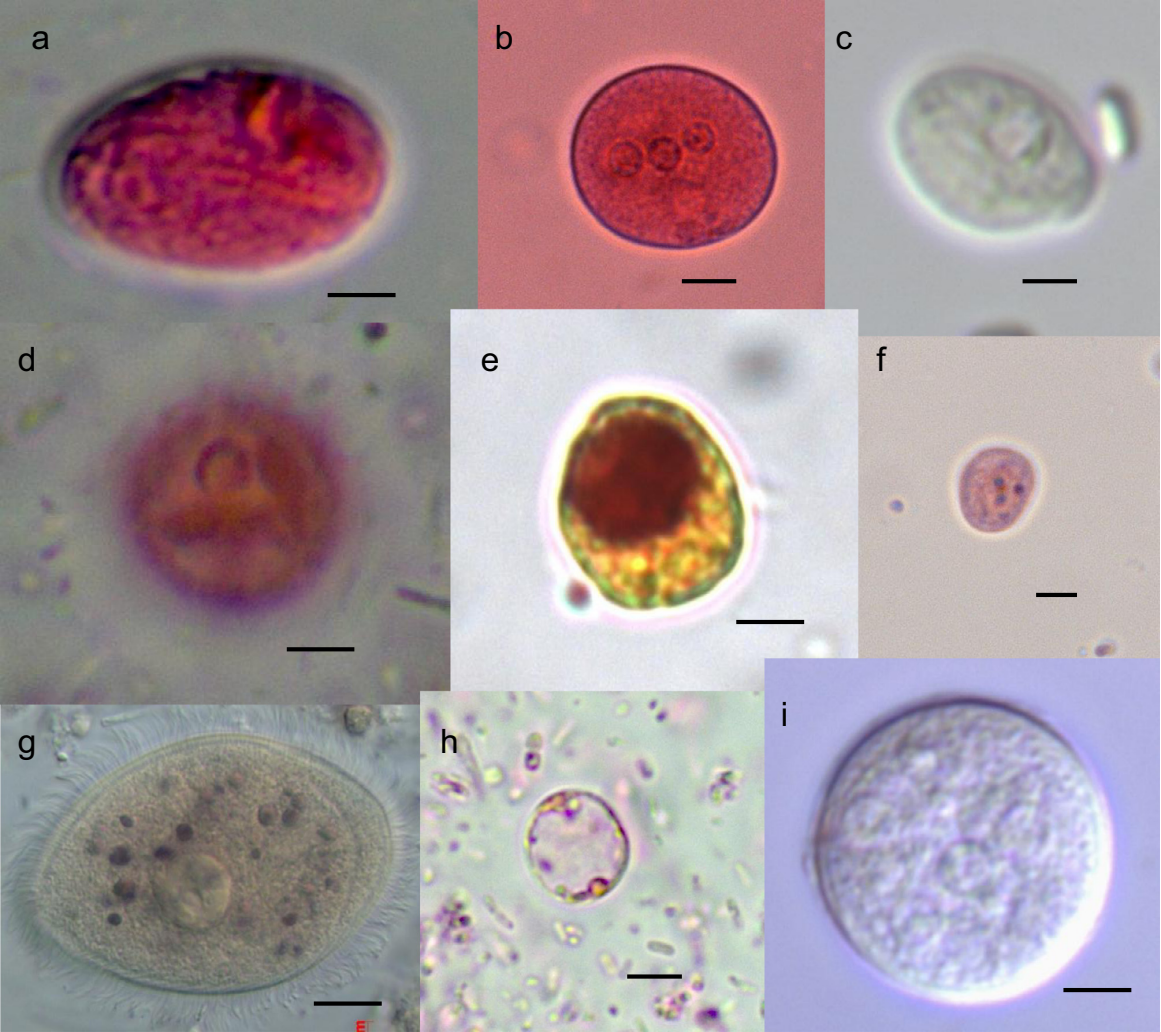
(a) *Giardia* sp. (cyst), (b) *Entamoeba hartmanni* (cyst), (c) *Chilomastix mesnili* (cyst), (d) *Entamoeba histolytica/dispar* (cyst), (e) *Iodameoba butschlii* (cyst), (f) *Endolimax nana* (cyst), (g) *Balantidium coli* (trophozoites), (h) *Blastocystis* sp. (cyst), (i) *Entamoeba coli* (cyst). Scale bars: a–i = 5 μm.

**Figure 3.**
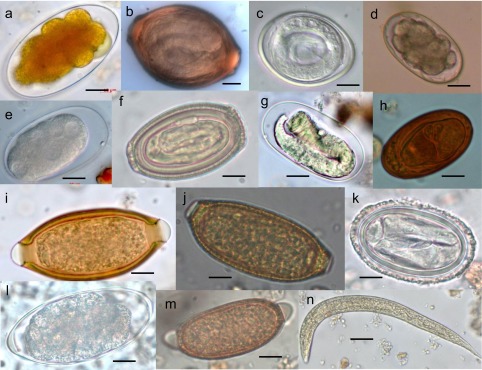
(a) *Ternidens* sp., (b) *Anatrichosoma* sp., (c) *Subulura* sp., (d) *Ancylostoma* sp., (e) *Oesophagostomum* sp., (f) *Chitwoodspirura* sp., (g) *Strongyloides* sp., (h) *Dicrocoelium* sp., (i) *Trichuris* sp., (j) Capillariidae Gen. sp. 1, (k) *Protospirura muricola*, (l) *Trichostrongylus* sp., (m) Capillariidae Gen. sp. 2, (n) *Strongyloides stercoralis* (rhabditoid larva). Scale bars: a–m = 10 μm; n = 25 μm.

Nine protozoa (Fig. 2) and 14 helminths (Fig. 3) were detected. All seven non-human primate species were infected by 9 protozoa (*Entamoeba coli*, *E. histolytica/dispar*, *E. hartmanni*, *Endolimax nana*, *Iodamoeba butschlii*, *Chilomastix mesnili*, *Balantidium coli*, *Blastocystis* sp., and *Giardia* sp.) and 5 helminths (*Oesophagostomum* sp., *Ancylostoma* sp., Capillariidae Gen. sp. 2, *Strongyloides* sp., and *Trichuris* sp.). The remaining nine helminths were distributed in the Taï monkeys as follows: Capillariidae Gen. sp. 1 and *Ternidens* sp. were found in *C. campbelli*, *C. diana*, and *C. petaurista. Subulura* sp. was detected in *C. campbelli* and *C. diana*. *Protospirura muricola* was identified in *C. petaurista* and *C. diana*. *Anatrichosoma* sp. and *Chitwoodspirura* sp. were revealed only in *C. petaurista*. *Trichostrongylus* sp. was detected in *C. campbelli* and *P. verus*. *Dicrocoelium* sp. was found in *P. badius*, *C. polykomos*, and *P. verus. Strongyloides stercoralis* was identified in *C. campbelli*, *C. diana*, *C. petaurista*, and *P. verus*.

### Diversity index and prevalence of parasite species in Taï monkeys

A total of 23 species of parasites were found in the seven monkey species at Taï. *C. atys* and *C. petaurista* were infected by 20 gastrointestinal parasites (86.96%). Nineteen species of parasites (82.61%) were detected in *C. campbelli* and *C. diana*. The lowest number of parasite species was recorded in *P. badius* and *C. polykomos* with 15 parasites (65.22%). For all parasites, species diversity as reflected by Shannon’s diversity index and equitability was high in all monkeys. However, the highest diversity and equitability indices were recorded in *C. diana* (*H* = 2.394; *E* = 0.8132), *C. petaurista* (*H* = 2.364; *E* = 0.7901), *Cercocebus atys* (*H* = 2.191; *E* = 0.7315), and *C. campbelli* (*H* = 2.159; *E* = 0.7332). For the helminths, Shannon’s index and equitability was higher with more equitable distribution in *C. diana*, *C. petaurista*, and *C. campbelli*. *Cercocebus atys* exhibit higher values (*H* = 1.902; *E* = 0.7934). Protozoa have higher Shannon index and high equitable distribution in *C. campbelli*, *C. diana*, *C. petaurista*, *P. badius*, *C. polykomos*, and *P. verus*. *Cercocebus atys* exhibit lower values (*H* = 0.8157; *E* = 0.3712) (Table 2).

**Table 2.** Parasite richness and Shannon’s diversity index.

Monkey species	Number of parasites	Parasite richness (%)	Diversity index (*H*)	Equitability index (*E*)
Helminths and protozoa				
*C. atys*	20	20/23 (86.96%)	2.251	0.718
*C. campbelli*	19	19/23 (82.61%)	2.173	0.7136
*C. diana*	19	19/23 (82.61%)	2.446	0.8035
*C. petaurista*	20	20/23 (86.96%)	2.387	0.7841
*P. badius*	15	15/23 (65.22%)	1.574	0.5812
*C. polykomos*	15	15/23 (65.22%)	1.3	0.4498
*P. verus*	17	17/23 (73.91%)	1.343	0.4647
Helminths				
*C. atys*	11	11/14 (78.57%)	1.902	0.7934
*C. campbelli*	10	10/14 (71.43%)	1.723	0.7484
*C. diana*	10	10/14 (71.43%)	1.887	0.8197
*C. petaurista*	11	11/14 (78.57%)	1.738	0.7246
*P. badius*	6	6/14 (42.86%)	1.092	0.6097
*C. polykomos*	6	6/14 (42.86%)	0.8384	0.4372
*P. verus*	8	8/14 (57.14%)	0.9013	0.4203
Protozoa				
*C. atys*	9	9/9 (100%)	0.8157	0.3712
*C. campbelli*	9	9/9 (100%)	1.946	0.8858
*C. diana*	9	9/9 (100%)	2.005	0.9124
*C. petaurista*	9	9/9 (100%)	1.991	0.9063
*P. badius*	9	9/9 (100%)	1.832	0.8337
*C. polykomos*	9	9/9 (100%)	1.859	0.8462
*P. verus*	9	9/9 (100%)	1.816	0.8264

The majority of gastrointestinal parasites found in the Taï primates exhibit relatively high abundances. *Entamoeba coli* (91.96%), *Balantidium coli* (60.3%), *Iodamoeba butschlii* (53.37%), and *E. histolytica/dispar* (53.27%) were the most prevalent protozoa, whereas the predominant prevalent helminths were *Trichuris* sp. (93.02%), *Oesophagostomum* sp. (79.9%), *Strongyloides* sp. (74.42%) and *Ancylostoma* sp. (73.87%).

Considering all parasites, there are significant differences in prevalence among the seven monkey species (*P* < 0.0001) (Table 3). Prevalence of individual parasites was compared only among monkey taxa infected by them and in most cases, there were statistically significant differences. *Anatrichosoma* sp. was detected in feces of *C. atys* (5.03%) and *C. petaurista* (3.18%). The difference in the prevalence of *Anatrichosoma* sp. was not statistically significant (*P* = 0.442). Differences in the prevalence of Capillariidae Gen. sp. 1 were significant between *C. campbelli* (13.48%), *C. petaurista* (3.18%), and *C. diana* (11.7%), (*P* = 0.004). Eggs of *Subulura* sp. were detected in *C. atys* (30.15%), *C. campbelli* (0.56%), and *C. diana* (2.92%). Differences in the prevalence of *Subulura* sp. were statistically significant (*P* < 0.0001). Differences in the prevalence of *Protospirura muricola* between *C. atys* (56.78%), *C. petaurista* (4.46%), and *C. diana* (9.36%) were significant (*P* < 0.0001). *Strongyloides stercoralis* was found in feces of *P. verus* (17.83%), *C. atys* (7.04%), *C. campbelli* (8.99%), *C. petaurista* (4.46%), and *C. diana* (2.34%). Differences in the prevalence of *Strongyloides stercoralis* were significant (*P* < 0.0001). Differences in the prevalence of *Trichostrongylus* sp. between *C. campbelli* (2.25%) and *P*. *verus* (5.43%) were not significant (*P* = 0.216). Dicrocoeliid eggs were identified in *C. atys*, *C. polykomos*, and *C. verus*. Differences in the prevalence of *Dicrocoelium* sp. in *C. atys* (36.68%), *C. polykomos* (32.8%), *P. badius* (15.51%), and *P. verus* (3.88%) were significant (*P* < 0.0001). *Ternidens* sp. was found in *C. atys* (9.55%), *C. campbelli* (8.43%), *C. petaurista* (2.55%), and *C. diana* (2.92%). Differences in the prevalence were significant (*P* < 0.0001).

**Table 3.** Prevalence (%) of gastrointestinal parasites in Taï forest monkeys.

		*C. atys n = 199*	*C. campbelli n = 178*	*C. diana n = 171*	*C. petaurista n = 157*	*P. badius n = 245*	*C. polykomos n = 125*	*P. verus n = 129*		
		Prevalence							*P*-value	Mean size (μm) ± 95% CI (μm)
Helminths	*Anatrichosoma* sp.	5.03	0	0	3.18	0	0	0	<10^−3^	72.33 × 52.79 ± 3.61 × 4.03
	*Ancylostoma* sp.	73.87	46.07	38.01	37.58	13.11	7.2	4.65	<10^−3^	65.64 × 41.4 ± 4.44 × 2.31
	Capillariidae Gen. sp. 1	0	13.48	11.7	3.18	0	0	0	<10^−3^	48.94 × 23.55 ± 2.05 × 0.99
	Capillariidae Gen. sp. 2	20.1	10.11	12.87	6.37	1.64	4	3.1	<10^−3^	57.73 × 23.88 ± 3.1 × 1.08
	*Chitwoodspirura* sp.	0	0	0	1.27	0	0	0	<10^−3^	40.4 × 23.8 ± 0.2 × 0.18
	*Oesophagostomum* sp.	79.9	56.18	25.15	30.57	15.92	8.8	4.65	<10^−3^	72.61 × 41.54 ± 1.61 × 2.78
	*Subulura* sp.	30.15	0.56	2.92	0	0	0	0	<10^−3^	41.68 × 33.84 ± 0.7 × 0.3
	*Strongyloides* sp.	33.17	21.35	21.64	21.02	31.84	14.4	74.42	<10^−3^	51.79 × 31.94 ± 4.27 × 2
	*Strongyloides stercoralis*	7.04	8.99	2.34	4.46	0	0	17.83	<10^−3^	254.7 × 17.2 ± 7.41 × 3.03
	Spirurid (cf. *Protospirura muricola*)	56.78	0	9.36	4.46	0	0	0	<10^−3^	54.97 × 37.37 ± 2.06 × 3.43
	*Ternidens* sp.	9.55	8.43	2.92	2.55	0	0	0	<10^−3^	90.08 × 50.31 ± 3.06 × 2.6
	*Trichuris* sp.	11.06	22.47	8.77	18.47	73.77	75.2	93.02	<10^−3^	57.78 × 26.7 ± 2.91 × 1.9
	*Trichostrongylus* sp.	0	2.25	0	0	0	0	5.43	<10^−3^	84.75 × 44.3 ± 3.77 × 0.9
	*Dicrocoelium* sp.	36.68	0	0	0	15.51	32.8	3.88	<10^−3^	40.15 × 22.28 ± 1.5 × 0.87
Protozoa	*Entamoeba coli*	91.96	58.43	60.82	79.62	59.43	49.6	61.24	<10^−3^	17.3 ± 2.1
	*Entamoeba histolytica/dispar*	53.27	28.09	28.07	43.95	23.77	13.6	29.46	<10^−3^	14.2 ± 1.2
	*Entamoeba hartmanni*	34.17	25.28	25.73	45.86	26.64	23.2	24.81	<10^−3^	10.3 ± 0.8
	*Endolimax nana*	16.58	24.72	26.9	36.31	17.62	12.8	13.95	<10^−3^	6.27 × 4.65 ± 0.86 × 0.44
	*Iodamoeba butschlii*	38.69	53.37	49.12	48.41	36.89	24.8	38.76	<10^−3^	14.26 × 10.37 ± 0.77 × 1.02
	*Balantidium coli*	60.3	4.49	4.68	6.37	8.61	4	11.63	<10^−3^	44.40 × 40.44 ± 3.26 × 4.28
	*Chilomastix mesnili*	32.66	14.61	26.9	31.21	19.67	10.4	20.93	<10^−3^	8.6 × 5.4 ± 0.83 × 1.1
	*Giardia* sp.	28.64	15.73	14.62	19.11	7.38	5.6	9.3	<10^−3^	11.96 × 8.99 ± 0.7 × 0.9
	*Blastocystis* sp.	37.19	7.3	9.36	9.55	4.92	4	8.53	<10^−3^	8.41 × 5.5 ± 0.82 × 0.53

## Discussion

Our analyses reveal significant richness and diversity of gastrointestinal parasites in the cercopithecid monkeys of Taï National Park. The diversity indices demonstrate considerable species diversity and an equitable distribution of gastrointestinal parasites found in these monkeys. By comparison, 21 gastrointestinal parasites were identified in Kenya’s Tana River mangabey [41] and 14 parasite species were identified in monkeys of Uganda’s Kibale Forest [18, 19]. Thirteen parasite species were found in Mahale National Park of Tanzania [30] and 12 species were found in Rubondo Island National Park of Tanzania [57]. Based on available data, the total of 23 gastrointestinal parasites recorded in the Taï monkeys represents the greatest parasite richness documented to date for African non-human primates.

A large majority of intestinal protozoa are reported with high prevalence in the Taï monkeys. These amoebae are common in non-human primates which are a well-known reservoir for gastrointestinal parasites due to a direct life cycle, and transmission by various forms of contact [56]. Amoebae were found in all seven primate taxa and relatively high prevalence was recorded. Several studies have highlighted amoebae in African non-human primates [18, 19, 26, 57]. *Entamoeba coli*, *E. histolytica/dispar*, and *Iodamoeba butschlii* had the highest prevalence in the Taï monkeys.

*Balantidium coli* is widely distributed in primates. *B. coli* is the only pathogenic ciliate and is the largest protozoa that parasitizes humans, but the risk for non-human primates has not been demonstrated [20]. We reported relatively low prevalence of *B. coli* in the Taï monkeys (11.63 to 4%) except for *Cercocebus atys* (60.3%). Survey of another African cercopithecid – the Tana River mangabey (*Cercocebus galeritus*) – revealed a lower prevalence of *B. coli* [41] than in our study. However, high prevalence was recorded in baboons of Mole National Park, Ghana [60].

Several studies have described the presence of *Blastocystis* sp. in wildlife, livestock, and humans [6, 55, 65, 71, 72]. A large number of morphological cell types have been described and the three major forms are vacuolar, granular, and amoeboid [9, 66, 74]. Nevertheless, *Blastocystis* sp. morphology is not well understood [12]. On the basis of molecular analysis of the ssrRNA gene, eight distinct subtypes (STs) ST1, ST2, ST3, ST4, ST5, ST8, ST13, and ST15 were isolated from non-human primates [1]. Vacuolar forms predominated in feces of the Taï monkeys.

*Giardia* sp. is a parasitic protozoan pathogen that infects the small and large intestines of a broad range of mammals including non-human primates. *G. duodenalis* ranges in clinical severity from asymptomatic to highly pathogenic. Trophozoites pass through the small intestine; they encyst and are then excreted with feces. Monkeys are infected by ingestion of *Giardia* cysts in food or water contaminated by feces [23]. High prevalence of *Giardia* sp. has been reported in wild *Colobus* in Ghana [67]. In many cases, high prevalence of *Giardia* infection has been recorded in primates living in disturbed forest or in forest fragments [61, 67]. Our findings demonstrate the relatively high prevalence of *Giardia* sp. in monkeys living in undisturbed forest. Molecular analyses will clarify the genotypes (assemblages) of *Giardia* sp. in these monkey populations.

*Oesophagostomum* sp. is one of the most common parasites identified in African primates [5, 11, 18, 19, 41, 60]. In humans, *Oesophagostomum* causes esophagostomosis resulting in the formation of granulomas, caseous lesions, or abscesses in intestinal walls; however, great apes are known to develop nodular esophagostomosis without the associated severe clinical signs [33] or associated morbidity and mortality [27]. A relatively high prevalence of infection is present in the Taï cercopithecids.

*Trichuris* sp. are parasitic nematodes that infect the ceca and colons of animal hosts and cause trichuriasis similar to that of humans. *Trichuris* sp. has a simple and direct life cycle. Similarities of *Trichuris* sp. infection are found in non-human primates and humans suggesting significant zoonotic transmission. However, many differences in egg morphometrics have been detected in non-human primates and the monkey-derived whipworm is a separate species from that found in humans [36]. Unfortunately, coprological analyses are inconclusive. A comprehensive study of genetic diversity is necessary to make a confident distinction between species. The *Colobus* species in the present study exhibited a very high prevalence, such as that found in primates of Boabeng-Fiema, Ghana, and Kibale Forest, Uganda [19, 67].

*Anatrichosoma* species are an unusual group of zoonotic trichuroid nematodes. Their occurrence is so rare that knowledge is limited and diagnosis uncommon [51, 53]. These rare nematodes have been identified in primates in Africa and Asia, but their life cycle remains unknown. Infections are normally recognized through the direct observation of adult worms from biopsy rather than through the recovery of eggs. Worms normally reside in the squamous epithelium of the nasal cavities [2, 38, 53]. Eggs are sloughed off and are usually swallowed where they are passed into the feces [53]. Eggs of *Anatrichosoma* in fecal samples were recently reported in chimpanzees and vervets of Tanzania’s Rubondo Island [57, 59]. In the present study, they were found in *Cercocebus* and *C. petaurista* which, to our knowledge, is the first report of *Anatrichosoma* sp. in *Cercocebus* and *Cercopithecus* species and the first in any free-ranging West African primate.

Capillariids are closely related to *Trichuris* and *Trichinella* species; all are members of the superfamily Trichinelloidea. Several species of the family Capillariidae are known to infect primates including humans: these are *Calodium hepaticum*, *Eucoleus aerophilus*, *Paracapillaria philippinensis*, *Aonchotheca brochieri*, and *A*. *annulosa* [14, 29, 47, 48]. Other species of Capillariids, such as those found in *Macaca*, were undetermined [29]. In the present study, we describe two distinct capillariids based on egg size and morphology. Study of adult worms combined with molecular analysis would provide for the determination of genus and species.

Hookworms are parasitic nematodes, belonging to the family Ancylostomatidae. They live in the small intestine of their hosts, which may be mammals such as humans and non-human primates [46, 54]. Infection with hookworms is due to the penetration of larvae (L3) through the skin or by direct ingestion of dirt (pica) or fresh vegetables containing filariform larvae. Infection via skin entry can cause skin inflammation and intestinal blood loss at the site of adult parasite intestinal attachment. The life cycle of hookworms is direct [25, 62]. Hookworms have been reported in several populations of free-ranging African primates [7, 20, 57, 58]. Differentiating the various hookworm species by morphological analysis of the eggs is difficult microscopically; however, the hookworms present in our samples appear to represent *Necator* sp. and/or *Ancylostoma* sp.

*Ternidens* spp. are widespread in African non-human primates (e.g., baboons and vervet monkeys) and are typically found in the large intestine of primates. They cause anemia and the development of nodules in the intestinal wall similar to those of *Oesophagostomum* spp. Because of the similarity between *Ternidens* eggs and those of hookworms and related species, microscopy diagnosis error is probably common. Thus, for identification purposes, measurement of egg size is important for differentiating hookworms and *T. deminutus*: hookworm eggs are approximately 70 μm × 40 μm while those of *T. deminutus* are approximately 84 μm × 51 μm [22]. *Ternidens* sp. have been found in *Cercopithecus* species [4, 63], such as those present in Taï National Park. A comprehensive molecular study is needed to more firmly establish species differences.

*Trichostrongylus* spp. are known to infect humans, wild animals, livestock and, especially, herbivorous animals. Primates can become infected due to environmental contamination by ruminants [49]. This study recorded *Trichostrongylus* only in *C. diana* and *C*. *verus* with low prevalence, in contrast to that found in baboons [52].

Non-human primates are known to be the major hosts of *Strongyloides stercoralis* and, especially, *S. fuelleborni*. Monkeys become infected by eating eggs or via skin penetration by third-stage infective larvae [17, 68]. Taï forest primates were infected by *Strongyloides stercoralis* and *Strongyloides* sp. However, *Strongyloides* sp. was predominant.

*Chitwoodspirura* sp. was described from adult worms collected in the stomach of gorillas [10]. The complements to the morphology of this species (worms and eggs) were provided later [50]. *Chitwoodspirura* is a spirurid nematode that has an arthropod intermediate host. The thick-shelled nature of the egg passed in the feces would suggest that the intermediate host is likely to be a coprophagous arthropod of some type that becomes infected by ingesting the embryonated egg. There is no known information about the life cycle of *Chitwoodspirura* sp. Eggs of *Chitwoodspirura* sp. were recorded recently in red colobus monkeys and in red-tailed monkeys in Tanzania [30]. At Taï, only *C. petaurista* were infected by *Chitwoodspirura* sp. and this is the first identified case in any West African primate.

The spirurid nematode *Protospirura muricola* is a parasite of African rodents. The life cycle requires an intermediate host and a synanthropic rat final host where it seems to be non-pathogenic [64]. In some primates, pathogenicity was variable and remains unknown in chimpanzees. *Protospirura muricola* has been reported in red-tailed monkeys and red colobus monkeys at Mahale National Park [30]. Egg morphology found in samples from the Taï monkeys matched descriptions of eggs found in Tanzanian chimpanzees [59].

*Dicrocoelium* spp. use insects (ants) as intermediate hosts [70]. Monkeys become infected by ingesting infected ants or food contaminated by ants. *Dicrocoelium* spp. (Trematoda, Dicrocoeliidae) live mainly in the bile ducts and gall bladders of domestic and wild ruminants and occasionally parasitize primates including humans. We have not yet recorded *Dicrocoelium* sp. in any *Cercopithecus* species from Taï; however, prevalence in the sooty mangabey and red colobus was similar to that found in Kenya’s Tana River primates [39].

## Conclusion

Our study provides baseline data on the gastrointestinal parasites in populations of free-ranging, forest-dwelling West African monkeys. More specifically, we provide the first parasite information on the cercopithecid monkey populations within Côte d’Ivoire’s Taï National Park. During our study, we recorded 23 gastrointestinal parasites with relatively high prevalence. No cestode species were recovered. Only coprological analyses based on morphological and morphometric examination were performed. Some identifications were made at the family or genus level; however, several parasites represent zoonoses. Further assessment of these via molecular analysis is underway which will allow us to not only examine incidences of cross-infection among these primates, but also evaluate the potential presence of zoonoses in this region. In addition to more thoroughly documenting the prevalence and diversity of parasite species, future research will facilitate better identification and understanding of the correlations between parasitism and diet, social behavior, home ranges, and group size in these and other primate taxa.
